# A rare presentation of urolithiasis in a child with urine retention from a nonendemic area: a case report and literature review

**DOI:** 10.1097/MS9.0000000000000301

**Published:** 2023-03-25

**Authors:** Joshua Muhumuza, Brian Musinguzi, Sedrick Bukyana, Dickson Kajoba, Stephen M. Kithinji, Selamo F. Molen

**Affiliations:** Departments of a Surgery; b Paediatrics and Child Health, Ishaka Adventist Hospital; Departments of c Paediatrics and Child Health; d Surgery, Faculty of Clinical Medicine and Dentistry, Kampala International University Western Campus, Ishaka Bushenyi; e Mubende Regional Referral Hospital, Mubende, Uganda

**Keywords:** case report, child, retention, stone, urethra, urolithiasis

## Abstract

Even though urolithiasis in general is not uncommon, urethral stones have an incidence of less than 0.3% and are 20 times less common in children. Though cases of urethral stones have been reported in children from endemic areas, they are even rarer in countries like Uganda that are not endemic for urolithiasis.

**Case Presentation:**

The authors present a 7-year-old male who presented with acute urine retention. Though the diagnosis of retention was made in a lower-level health facility, the cause of the retention was not determined till the patient arrived at a general hospital. Diagnosis of an obstructing stone in the penile urethra was made clinically. Meatotomy and stone extraction were done, and a urethral catheter was passed.

**Clinical Discussion and Conclusion:**

When attending to children with acute urine retention, urolithiasis should be kept among the differential diagnoses, even in areas that are not endemic for urinary tract stones. A thorough clinical evaluation may be all that is needed to make a diagnosis.

## Introduction

Highlights

- Urethral urolithiasis can present as acute urine retention, even in children.
- In areas not endemic for urinary tract stones, a high index of suspicion is necessary to make the diagnosis.
- A thorough clinical evaluation may be all that is needed to make a diagnosis.

Even though urinary tract stones are a relatively common problem, urethral stones are a rare type of urolithiasis, accounting for ~1% of all presentations^1^. Penile urethral stones are much rarer, with an incidence of less than 0.3%^1^, and even so, 20 times rarer in children^2^. The presence of an obstructing urethral stone causing acute urine retention is a urology emergency requiring immediate intervention^3^. Urethral stones are more common in males and usually originate in the upper tract or urinary bladder before travelling into the urethra, where they cause obstruction^4^,^5^. In some cases, native urethral stones form in the urethra and are associated with strictures, urethral diverticula, chronic infection, and urethral foreign bodies^6^. The majority of urethral calculi are usually calcium oxalate (85%)^7^.

Urinary tract stones have been reported to have a geographical pattern, and paediatric urolithiasis is said to be more common in places along the ‘stone belt’ that runs from the Philippines, Thailand, and Myanmar in the Far East through Pakistan into Iran, Middle East and extending up to Turkey^8^. In Uganda, a country outside the belt, reports on paediatric urolithiasis are hard to come by, let alone urethral urolithiasis presenting with acute urine retention. We present a rare case of urolithiasis in a child who presented with acute urine retention from a nonendemic area that was managed at a general hospital in a poor resource setting and has been reported in line with the SCARE 2020 criteria^9^.

## Case presentation

We present a 7-year-old male who had been completely well until 2 days prior to the presentation. He presented with a history of inability to pass urine, associated with lower abdominal pain, penile pain when attempting to pass urine, and dribbling. The mother reported no history of such symptoms in the past, and there was no significant medical or family history. Prior to presentation, the child had been managed in a peripheral lower-level health facility, where suprapubic aspiration of urine was done to temporarily relieve the abdominal pain given that urethral catheterisation had failed. The underlying cause was not ascertained at the lower-level health facility, and the failure to pass urine persisted, resulting in a referral to a general hospital for further evaluation and management.

**Figure 1:**
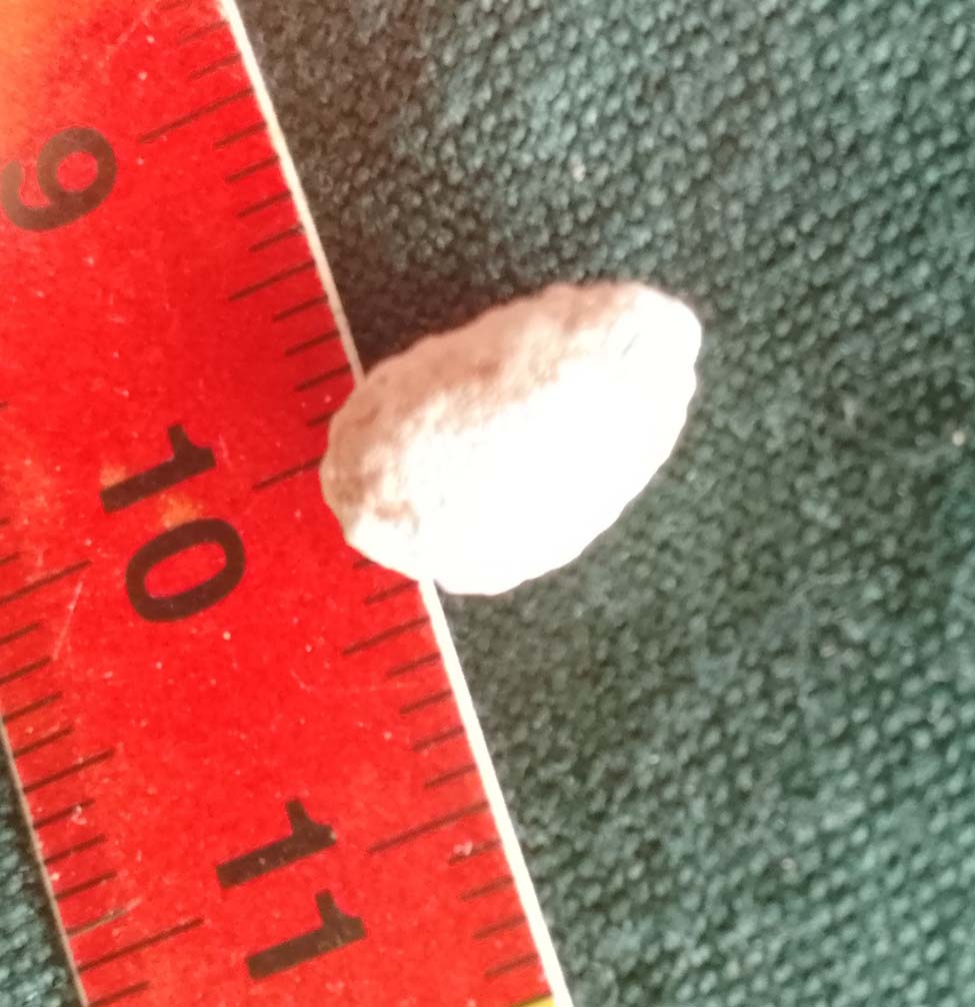
The stone after extraction.

**Figure 2:**
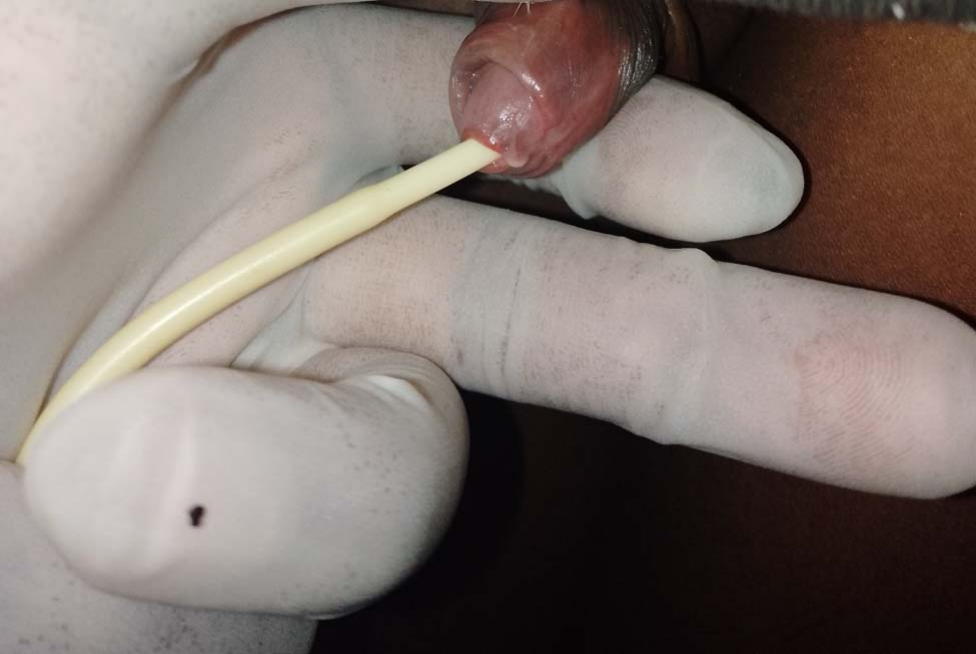
The catheter *in situ* after extracting the stone.

On examination, he was alert, with a tachycardia of 90 beats per minute and dry mucous membranes, but the rest of the vital signs were in the normal range. On abdominal examination, we noted a distention in the suprapubic region that was dull on percussion. While attempting to pass a urethral catheter, we noted that there was a whitish mass at the urethral meatus that was hard on palpation and could not be expressed out through the meatus. A working diagnosis of acute urine retention due to an obstructing stone in the urethral meatus was made. Under sedation, a stone measuring 11 by 7 mm (Fig. 1) was extracted by a general surgeon following a meatotomy, and a urethral catheter was passed (Fig. 2).

After relieving the urine retention, urinalysis revealed the presence of gram-negative rods, the complete blood count was unremarkable, and erythrocyte sedimentation rate was 29.4 mm/h and an abdominal radiography did not show any other stones in the urinary tract. Ultrasonography did not reveal any anatomical abnormalities. The patient was rehydrated, and oral cephalexin plus paracetamol were administered for 5 days. The catheter was removed on the fifth day, and the child was discharged with advice on adequate fluid intake. For the 2 months of follow-up at the general hospital, the child was well, and the mother reported no symptoms related to urolithiasis.

## Discussion

A urethral stone can lodge at any point of the urethra^10^, however the commonest site of obstruction is the prostatic region^7^. In this child, the obstruction was in the penile urethra at the meatus, and this made it easy for us to make a diagnosis. The aetiology of urolithiasis in the paediatric population is anatomical abnormality in 12% of the children, metabolic defects in 25%, urinary tract infections in 7%, and idiopathic in the majority (55%)^11^. In this child, gram-negative rods were found in the urine, which could suggest urinary tract infection as a cause, but also the mother reported that the child does not pack water when going to school, and this could also suggest inadequate fluid intake in addition to the fact that the child had features of dehydration at presentation. Ultrasonography did not show any anatomical abnormalities, but a computed tomography scan, which could have shown more details, was not available at the facility.

Patients usually present with dysuria or acute urine retention with a palpable stone in the external meatus or in the penile part of urethra^12^. Patients may also report haematuria, urinary incontinence, interrupted streams, weak stream with dribbling, perineal pain, rectal pain, or pain in the external meatus and urethra^13^–^15^. In this patient, only urine retention, penile pain on attempting to pass urine, dribbling, and a palpable urethral mass were noted. Abdominal and pelvic radiography or a retrograde urethrogram usually confirm the diagnosis^13^,^16^. A computed tomography scan and magnetic resonance imaging yield additional anatomical information and more details about the upper urinary tract that are essential for the diagnosis and management^17^. Untreated urethral calculus can cause urethral diverticulum, urethral abscess, and urethrocutanous fistula^18^. In this case, the diagnosis was made clinically since the child presented with features of acute urine retention in addition to the stone being visible at the meatus and palpable through the urethra.

The management options for urethral stones are variable and depend largely on various factors like the location of stone impaction, the size of the stone, and the presence or absence of any associated urethral pathologies. Retrograde manipulation of the stones back into the urinary bladder may be suitable in patients with small urethral stones, followed by litholapaxy or lithotripsy^15^. Anterior urethral stones may be extracted by means of endoscopic removal or by ventral meatotomy when the stone is impacted in the distal shaft of the penis^19^. Since initial attempts at extracting the stone by lubrication alone were futile, a meatotomy was done.

Even in areas like western Uganda that are not endemic for urolithiasis, when children present with acute urine retention, the attending clinician needs to keep urinary tract stones on the list of possible causes. More so, examination of the urethra should not be ignored since this can help in making a diagnosis and giving definitive care. When attempts to pass a urethral catheter are futile, thorough examination of the urethra by palpation may help to find the stone.

## Conclusion

Clinicians attending to children presenting with acute urine retention need to keep in mind that the cause could be urolithiasis, even in regions that are not endemic for urinary tract stones. A thorough examination of the urethra may be all that is required to make the diagnosis.

## Ethical approval

Not applicable.

## Consent for publication

We obtained written informed consent from the mother of the child for the publication of the case report and the images. A copy of the consent is available for review by the editor in chief of this journal on request.

## Sources of funding

This case report did not receive any specific grant from funding agencies in public, commercial, or not for profit sectors.

## Author contribution

J.M. managed the patient and wrote the first draft. B.M. and S.B. were involved in patient management, review and editing of the manuscript. D.K., S.M.K., and S.F.M. reviewed and edited the manuscript. All authors approved the final version of the manuscript submitted.

## Conflicts of interest disclosure

None.

## Research registration unique identifying number (UIN)

1. Name of the registry: not applicable.
2. Unique identifying number or registration ID: not applicable.
3. Hyperlink to your specific registration (must be publicly accessible and will be checked): not applicable

## Guarantor

Joshua Muhumuza.

## Provenance and peer review

Not commissioned, externally peer-reviewed.
